# Advancing ASD identification with neuroimaging: a novel GARL methodology integrating Deep Q-Learning and generative adversarial networks

**DOI:** 10.1186/s12880-024-01360-y

**Published:** 2024-07-25

**Authors:** Yujing Zhou, Guangbo Jia, Yingtong Ren, Yingxin Ren, Zhifeng Xiao, Yinmei Wang

**Affiliations:** 1 Center for Sleep and Circadian Medicine, The Affiliated Brain Hospital, Guangzhou Medical University, Guangzhou, 510370 Guangdong China; 2 Key Laboratory of Neurogenetics and Channelopathies of Guangdong Province and the Ministry of Education of China, Guangzhou Medical University, Guangzhou, 510260 Guangdong China; 3 Shenzhen Mental Health Center & Shenzhen Kangning Hospital, Shenzhen, China; 4 Biomedical Engineering, Northeastern University, Shenyang, China; 5 Automation, Northeastern University, Shenyang, China; 6 China Nanhu Academy of Electronics And Information Technology, Jiaxing, China; 7 Psychiatric Department of The Second Affiliated Hospital, School of Medicine, The Chinese University of Hong Kong, Shenzhen and Longgang District People’s Hospital of Shenzhen, Shenzhen, 518172 China

**Keywords:** Autism spectrum disorder, Generative adversarial networks, Deep Q-learning network, Data augmentation, Functional magnetic resonance imaging, Biomarkers

## Abstract

Autism Spectrum Disorder (ASD) is a neurodevelopmental condition that affects an individual's behavior, speech, and social interaction. Early and accurate diagnosis of ASD is pivotal for successful intervention. The limited availability of large datasets for neuroimaging investigations, however, poses a significant challenge to the timely and precise identification of ASD. To address this problem, we propose a breakthrough approach, GARL, for ASD diagnosis using neuroimaging data. GARL innovatively integrates the power of GANs and Deep Q-Learning to augment limited datasets and enhance diagnostic precision. We utilized the Autistic Brain Imaging Data Exchange (ABIDE) I and II datasets and employed a GAN to expand these datasets, creating a more robust and diversified dataset for analysis. This approach not only captures the underlying sample distribution within ABIDE I and II but also employs deep reinforcement learning for continuous self-improvement, significantly enhancing the capability of the model to generalize and adapt. Our experimental results confirmed that GAN-based data augmentation effectively improved the performance of all prediction models on both datasets, with the combination of InfoGAN and DQN's GARL yielding the most notable improvement.

## Introduction

ASD is a developmental impairment characterized by limited and repetitive behavioral patterns and impaired social communication [1]. ASD is a collective term for a number of pervasive neurodevelopmental conditions that can pose serious social, communicative, and behavioral difficulties, raising serious public health issues. The devastating impact of ASD is that it impacts the parents, siblings, and other family members in addition to the child., as well as disturbing the daily life of the affected family. Early identification of ASD can lead to early treatment and better outcomes. In recent studies, several instruments and techniques have been created for the early detection and characterization of ASD characteristics. Neuroimaging methods are widely considered to be effective for the proper characterization of ASD and early diagnosis [2].

In order to diagnose and treat brain-based diseases early on, psychiatric neuroimaging research is working to identify objective biomarkers Meanwhile, neuroimaging data analysis using machine learning and deep learning techniques has shown promise for locating people with psychiatric and neurological illnesses [3]. The evaluation of social behaviors and linguistic abilities is necessary for the diagnosis of ASD. The complexity of the range of behavioral changes seen in people with autism and their neurological patterns are linked, according to neuroscientific studies [4]. Understanding the neural bases of ASD and related social and communication difficulties is also aided by non-invasive brain imaging research [5–7], and even larger brain-based diseases and their related behavior [8] discovered the patterns of activation for ASD and the linkage of the patterns with neurological and psychological components that helped understand the etiology of mental disorders.

Hiremath et al. provided a systematic review [2] of recent findings for early diagnosis, ASD may benefit from early intervention and have a better outcome. They focus on early biomarkers for the characterization of ASD features at a younger age, using behavioral and quantitative MRI methods. For feature extraction and classification tasks, machine learning algorithms offer adequate methods for properly evaluating ASD. The use of machine learning techniques in the field of ASD often involves two types of approaches: conventional techniques [9] and deep learning techniques [10]. Compared to conventional methods, substantially less research has been done on DL methods [3], which use deep learning algorithms to identify patients' brain activation patterns indicative of autism spectrum disorder by merging a multilayer perceptron (MLP) and autoencoders (ASD). ABIDE (Autism Brain Imaging Data Exchange) I served as the dataset for an investigation on functional connectivity patterns that distinguish ASD patients from healthy controls in functional brain imaging. The algorithm obtained 70% mean classification accuracy utilizing connectivity characteristics derived from the Craddock 200 (CC200) brain atlas. The paper also determined the regions of the brain that were primarily responsible for recognizing ASD using deep learning techniques. Nonetheless, the method required extensive training (more than 32 h).

In recent studies, Xu et al. [11] utilized electroencephalogram (EEG) signals and a combined Convolutional Neural Network (CNN) and Long Short-Term Memory (LSTM) model for diagnosing ASD. Their method enhances the diagnostic accuracy of ASD by analyzing brain functional connectivity through time series maps. Wang et al. [12] proposed a multimodal ASD diagnosis method based on the Deep Graph Convolutional Network (DeepGCN), which utilizes functional MRI data and demographic information to complement the classification task of diagnosing subjects. Furthermore, Wang et al. [13] introduced a deep learning framework for ASD diagnosis using multimodal data while considering privacy preservation. Their method employs Hypergraph Neural Networks (HGNN) and Federated Learning to integrate functional neuroimaging data with personal characteristic data, capturing the interrelationships within multimodal data.

In this study, we use resting-state functional magnetic resonance imaging (rs-fMRI) data to categorize participants with and without ASD based on the different brain patterns of functional connectivity. Recent advances have witnessed the extensive usage of rs-fMRI data to build machine-learning models for ASD identification. The Autism Imaging Data Exchange (ABIDE) program has made a significant contribution to the global collaboration of numerous research centers. Over a thousand samples have been gathered and added to the dataset and have been utilized by numerous prior studies. However, from a deep learning perspective, a little over a thousand examples are not sufficient to train a robust model to capture a rich set of features. Therefore, underfitting may happen, leading to a relatively high bias and low accuracy. Earlier research has looked into a number of ways to increase model-side detection accuracy. In other words, a wide range of learning algorithms have been validated on this dataset. However, few efforts have explored the potential of data augmentation, which could be another effective venue to boost detection accuracy. By introducing generative data augmentation approaches into the learning pipeline, we hope to close this gap in our work. The proposed method can learn patterns from existing training data in an unsupervised fashion and generate synthetic samples, which form an augmented training set that also participates in model training, alongside the original training set. The augmented samples are similar to the real samples and can effectively increase the diversity of training data, which benefits the training. To our knowledge, using generative models for data augmentation for ASD detection has not been seen in the literature. In summary, the core contributions are twofold. First, we propose to utilize generative models for data augmentation in the ASD detection problem. Three generative adversarial network (GAN) models are being used to increase the size and variety of the training set. We then train five predictive models using the augmented dataset and evaluate their scores on the test set. Findings show that models trained on the enhanced data—including those trained using the SOTA method—have consistently outperformed models based on the original training set. This validates the efficacy of the suggested approach.

This research proposes an innovative data augmentation method based on GANs for early diagnosis of ASD. This method effectively expands the dataset for ASD diagnosis by generating synthetic samples that capture the distribution of the original functional Magnetic Resonance Imaging (fMRI) data. Not only does it increase the amount of training data, but it also enhances data diversity, thereby improving the robustness and generalization ability of the diagnostic model. The main contributions include:

1. Proposing the use of GANs for data augmentation, expanding the scale and diversity of the ASD fMRI dataset to facilitate early and precise diagnosis.
2. Exploring three GAN models (DCGAN, WGAN, and InfoGAN) for data augmentation and evaluating their performance on the ASD dataset.
3. Combining GAN data augmentation with various advanced deep learning and machine learning models, and validating the effectiveness and robustness of this method through comparative experiments, significantly improves the accuracy, sensitivity, and specificity of ASD diagnosis.
4. Investigating the combination of the Underlying Knowledge-based Semi-Supervised Learning (UKSSL) [4] technique with GANs to leverage unlabeled data and medical domain knowledge, further enhancing diagnostic performance.
5. Inspired by recent applications of weakly supervised machine learning [14] and deep learning food category recognition [15] and other domains, demonstrating the versatility and potential of these advanced methods in the medical field of ASD diagnosis.
6. Showcasing the application prospects of artificial intelligence methods such as GANs in medical imaging and clinical data analysis, providing powerful solutions to address challenges like data scarcity in biomedicine, and contributing to the advancement of precision medicine and personalized treatment.

## Materials and methods

### The abide dataset

Nowadays more than 1% of children are diagnosed with ASD, characterized by repetitive, restricted, and stereotyped behaviors/interests, as well as qualitative impairment in social reciprocity. It becomes an urgent need for diagnosis at earlier ages to select optimal treatments and predict outcomes. However, The urgency has not been met by the state of current research or its clinical implications. Due to the complexity and heterogeneity of ASD, large-scale samples are essential to reveal the brain mechanisms underlying ASD, which cannot be achieved by a single laboratory. To address this issue, Data on structural and functional brain imaging have been gathered by the ABIDE program from laboratories all around the world, which advances the research on the neural bases of autism. There are currently two large-scale collections in ABIDE: ABIDE I and ABIDE II, which are openly accessible to the globe and independently gathered across more than 24 international brain imaging laboratories, which are independently collected across more than 24 international brain imaging centers and available to researchers worldwide.

The initial ABIDE program, ABIDE I, featured 17 international locations sharing previously gathered anatomical, phenotypic, and resting-state functional magnetic resonance imaging (R-fMRI) datasets. It was released in August 2012 with 1112 datasets (539 from individuals with ASD and 573 from typical controls) being shared alongside the larger scientific community. The popularity of these data usage and resulting publication proved its value for examining both whole-brain and local characteristics in ASD. All datasets are anonymized without any privacy issues.

Due to the complexity of the connectome, research results from ABIDE I data analyses indicate considerably bigger and more complete samples. ABIDE II was established with funding from the National Institute of Mental Health to advance research on the brain connectome in ASD. ABIDE II has gathered approximately 1,000 new datasets with improved phenotypic characterization as of March 2017 (the most current update), especially connected to measurements of the basic symptoms of ASD. ABIDE II was released for public research in June 2016, with 19 sites involved, 1114 datasets from 521 people with ASD, and 593 controls donated. Similar to ABIDE I, all datasets in ABIDE II are anonymous, with no privacy issues. In this research, we choose ABIDE II as our dataset. Table 1 shows the participants' phenotypic data summary from the ABIDE II dataset.

**Table 1:** Phenotypic information summary of the participants from the ABIDE II dataset

Site	ASD(#)	Control(#)	Total	Age Range
BNI	29	29	58	18–64
EUMCR	27	27	54	6–11
ETH	13	24	37	14–31
GU	51	55	106	8.1–13.9
IU	20	20	40	17–54
IPRDH	22	34	56	6–47
KUL	28	0	28	18–35
KKI	56	155	211	8–13
NYULMC1	48	30	78	5.2–34.8
NYULMC2	27	0	27	5.1–8.8
ONRC,ILHH	24	35	59	18–31
OHSU	37	56	93	7–15
TCHS	21	21	42	10–20
SDSU	33	25	58	7.4–18
Stanford	21	16	42	8–13
UCDavis	18	14	32	12–17
UCLA	16	16	32	8–15
U.Miami	13	15	28	7–13
U.UtahSM	17	16	33	9–39
UCLA Longitudinal	14	7	21	8–15
UPSM Longitudinal	9	8	17	9–18

Preprocessing: Pipeline methods are used in ABIDE data processing, which involves generic preprocessing procedures. There are four pipeline strategies used to preprocess ABIDE datasets: the neuroimaging analysis kit (NIAK) [16], the configurable pipeline for the analysis of connectomes (CPAC) [17], the connectome computation system (CCS), or the data processing assistant for rs-fMRI (DPARSF) [18, 19]. Different pipelines carry out comparatively similar preprocessing steps. The parameters, program simulations, and individual algorithm steps are where there are the biggest disparities. The specifics of each pipeline approach are described in [20].

### System framework

**Fig. 1:**
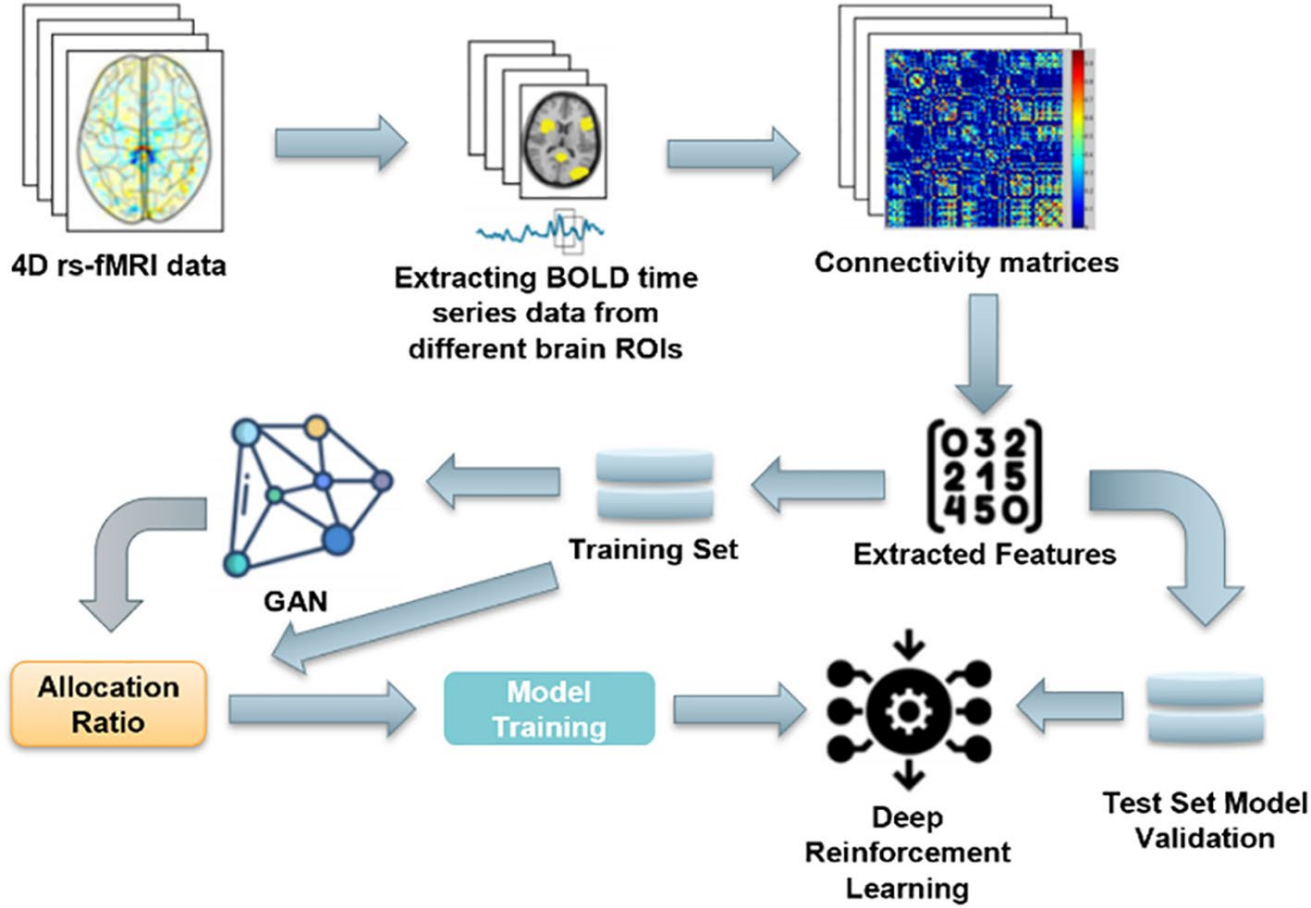
GARL Framework: Advancing ASD Diagnosis with GAN-Enhanced rs-fMRI Analysis. This process provides an overview of the proposed GARL approach for ASD analysis from resting-state functional MRI (rs-fMRI) data

Figure 1 illustrates the learning pipeline of the proposed system. Initially, the collected 4D rs-fMRI data is processed to extract the BOLD time series from various brain regions of interest (ROIs). These time series data are analyzed to identify co-activated pairwise ROIs, forming connections between regions. These connections are recorded in a matrix, which is subsequently flattened into a feature vector, representing a patient sample. These feature vectors are divided into a training set and a test set.

The training set is used to train a Generative Adversarial Network (GAN) that captures the inherent patterns in the data. The GAN-generated synthetic data, combined with real data, forms an augmented dataset. This augmented dataset is then used to train a reinforcement learning (RL) network to assist in ASD diagnosis. The RL network is guided by a reward function that incentivizes accurate diagnoses and penalizes errors.

The predictive models, trained on this augmented dataset, can make diagnostic decisions based on both real and synthetic data. These decisions include diagnosing ASD and recommending treatment plans. The performance of these models is evaluated using the test set to determine their final prediction accuracy. In summary, the modified content would describe the new learning pipeline, including the use of a GAN and RL network to diagnose ASD and the steps involved in gathering and processing the data.

### GAN-based data augmentation

A GAN belongs to a class of machine learning frameworks that may generate fresh data with the same statistics after learning the sample distribution from a given dataset. In this study, we generate fresh training samples based on the original dataset using a GAN-based data augmentation strategy. The freshly created data samples improve the quality and diversity of the original dataset by having distributions that are somewhat similar, if not exactly the same, as those in the original dataset. In this study, the information-maximizing GAN (InfoGAN) [21], the conditional GAN (cGAN), and the vanilla form of GAN are the three types that we are primarily interested in.

### Dynamic reinforcement learning based on vanilla GAN

**Fig. 2:**
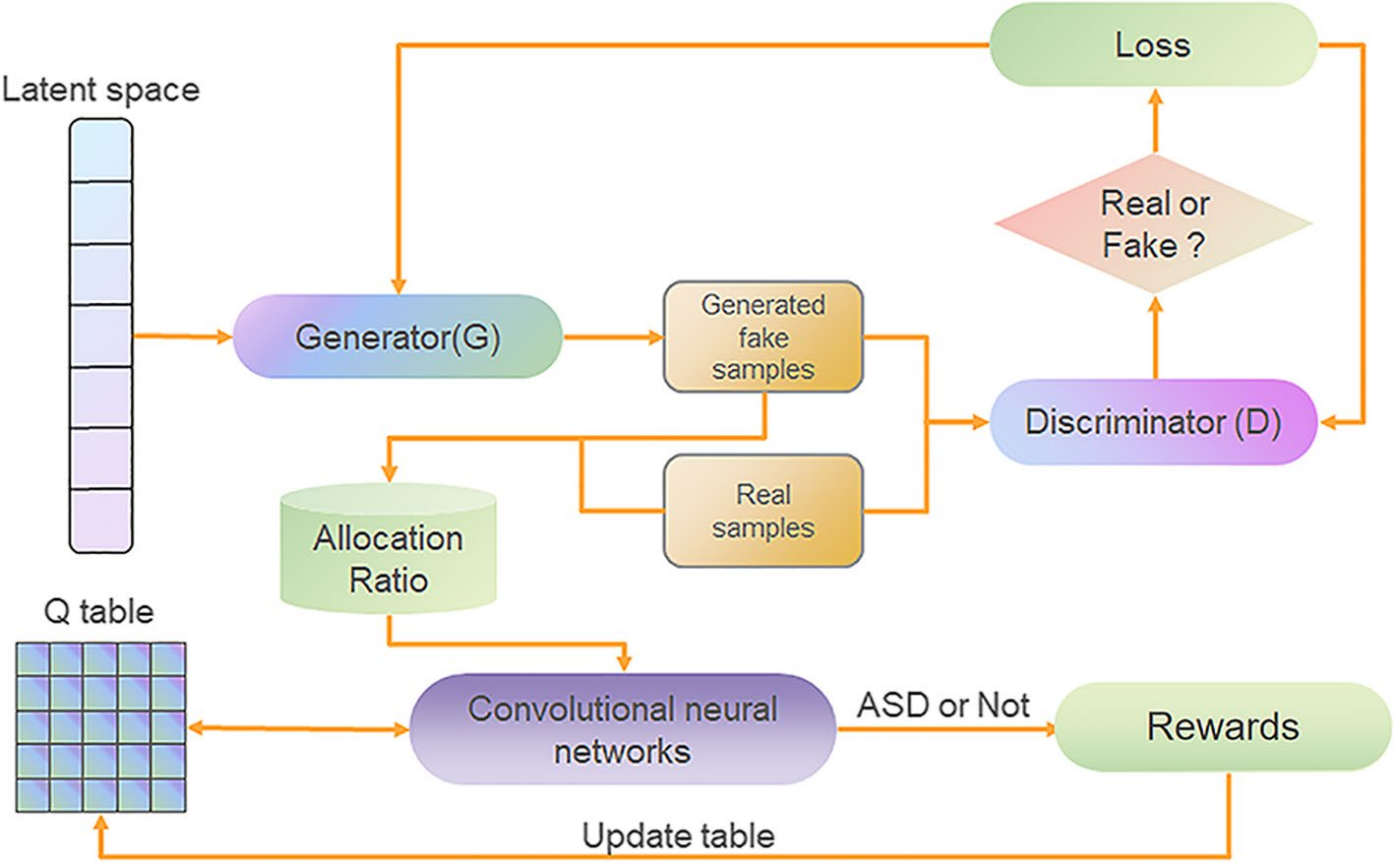
Enhanced GAN Framework for ASD Detection with Deep Q-Learning. This diagram depicts an advanced Generative Adversarial Network (GAN) framework integrated with Deep Q-Learning for improved detection of ASD from complex data inputs. It encapsulates a dynamic interaction between a generator producing synthetic data and a discriminator evaluating authenticity, fine-tuned by reinforcement learning to optimize diagnostic decisions

As shown in Fig. 2, the vanilla GAN is used as the environment to generate virtual ASD data to increase the number of samples to help train the reinforcement learning network. the generator of the vanilla GAN receives a random vector as input and then generates virtual data. The discriminator receives both real and virtual data and then outputs a scalar indicating whether the input data is real or virtual. The generator and discriminator play against each other, with the expectation that the generator will generate more realistic virtual data and the discriminator will be able to accurately distinguish between real and virtual data.

Let G and D represent the generator and discriminator, respectively, in formal terms; let z stand for the random vector, and G(z) for the artificial sample that was created, which is very similar to a real sample, *x*; let $\{\left({\text{x}}_{\text{i}},1\right){\}}_{\text{i}=1}^{\text{m}}$ and $\{\left(\text{G}\left({\text{z}}_{\text{i}}\right),0\right){\}}_{\text{i}=1}^{\text{m}}$ be the sets of identified false and actual samples, each with m samples, together being used to train the discriminator D, which produces a binary output D(*x*), predicting a real (D(x) = 1) or fake (D(*x*) = 0) result. The discriminator is honed to maximize both the accuracy of real and fictitious sample predictions. To put it another way, if an input sample x is real, D works to move D(x) in the direction of 1, which leads to a successful prediction, and vice versa. Equation 1 presents the optimization issue for D. Similar to Eq. 2, generator G seeks to reduce D's ability to identify G(z) as a forgery. Combining the two optimization issues, we arrive at a MinMax game that is formulated in 3. The MinMax game can be solved using an equilibrium by a GAN, which is possible when both G and D are sufficiently optimized and converge.

1 $$\underset{D}{\max}V\left(D\right)={E}_{x\sim {P}_{data}\left(x\right)}\left[\log\ D\left(x\right)\right]+{E}_{z\sim {p}_{z}\left(z\right)}\left[\log\left(1-D\left(G\left(z\right)\right)\right)\right]$$

2 $$\underset{\text{G}}{\min}\text{V}\left(\text{G}\right)={E}_{z\sim {p}_{z}\left(z\right)}\left[\log\left(1-\text{D}\left(\text{G}\left(\text{z}\right)\right)\right)\right]$$

3 $$\underset{G}{\min}\ {\underset{D}{\max}}V\left(D,G\right)={E}_{x\sim {p}_{data}\left(x\right)}\left[\log\ D\left(x\right)\right]+{E}_{z\sim {p}_{z}\left(z\right)}\left[\log\left(1-D\left(G\left(z\right)\right)\right)\right]$$

The training procedure, which involves several epochs, is described in the sections that follow. Each epoch consists of multiple time steps. For every time interval, the first select many random vectors $\{{z}_{i}{\}}_{i=1}^{m}$ from the noise prior and sampling a collection of actual samples $\{{x}_{i}{\}}_{i=1}^{m}$ from the training set; Then, by increasing its stochastic gradient, we update the D${\Delta }_{{\Theta }_{d}}\frac{1}{m}{\sum }_{i=1}^{m}\left[\log D\left({x}_{i}\right)+\log\left(1-D\left(G\left({z}_{i}\right)\right)\right)\right]$. After k time steps, we choose a new set of random vectors. $\{{z}_{i}{\}}_{i=1}^{m}$, along with updating G by lowering its stochastic gradient${\Delta }_{{\Theta }_{g}}\frac{1}{m}{\sum }_{i=1}^{m}\log\left(1-D\left(G\left({z}_{i}\right)\right)\right)$. D and G are then successively optimized till convergence.

Using Deep Q-learning as the structure for reinforcement learning, the network receives the environment (i.e. virtual data generated by the GAN) and real data as input. The output of the network is a set of actions, including diagnosing whether a patient has ASD or recommending a treatment plan. The network maximizes the cumulative reward by selecting different actions in different states. The network continuously updates its strategy to achieve higher rewards in subsequent states.

In Rewords, if the agent diagnoses an ASD patient as ASD, the reward is 1. If the agent misdiagnoses a non-ASD patient as ASD, the reward is -1. In all other cases, the reward is 0. Through this reward function, the agent can learn how to identify ASD patients as accurately as possible during the diagnosis Through this reward function, the agent can learn how to identify ASD patients as accurately as possible during the diagnosis process.

The overall dataset is D, which contains the real sample ${D}_{real}$ and the vanilla GAN-generated sample ${D}_{fake}$, with ${D}_{real}$ and ${D}_{fake}$ of size ${N}_{real}$ and ${N}_{fake}$ respectively. We use a weight parameter λ to balance the use of these two types of data, where $0\le \lambda \le 1$. When λ = 0, only real data is used for training; when λ = 1, only vanilla GAN-generated data is used for training.

The recursive process transforms the scale of the data set by gradually increasing λ. A total of k iterations are required, using λ for each iteration as ${\lambda }_{1},{\lambda }_{2},\cdots ,{\lambda }_{K}$, where ${\lambda }_{1}$=0 and ${\lambda }_{k}$=1. In the kth iteration, the data set used is ${D}_{k}=(1-{\lambda }_{k}){D}_{real}+{\lambda }_{k}{D}_{fake}$. Specifically, the proportion of real samples and vanilla GAN-generated samples in D_k can be calculated using Eq. 4 and Equation.

4 $$\frac{{N}_{real,k}}{{N}_{real,k}+{N}_{fake}}=1-{\lambda }_{k}$$

5 $$\frac{{N}_{fake,k}}{{N}_{real,k}+{N}_{fake}}={\lambda }_{k}$$

where ${N}_{real,k}$ denotes the number of real samples used in the kth iteration and ${N}_{fake,k}$ denotes the number of vanilla GAN-generated samples used.

During the training process, a small batch of samples is randomly sampled from ${D}_{k}$ at each iteration based on the current ${\lambda }_{k}$ for training. As ${\lambda }_{k}$ gradually increases, the vanilla GAN-generated samples gradually take up a larger proportion, and thus the trained model gradually adapts to the vanilla GAN-generated data distribution.

The reinforcement learning used in all the later GAN structures is the same as this.

### InfoGAN

**Fig. 3:**
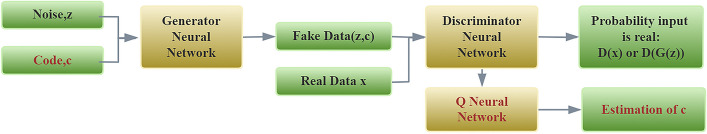
InfoGAN: Disentangling Data Semantics with Information Maximizing GAN. This schematic elucidates the InfoGAN structure, an advanced variation of the traditional GAN that enhances data interpretation by infusing a latent code into the generative process, offering a more discernible data synthesis

In the vanilla GAN, the input noise may be used in a highly entangled manner without being constrained in any way by the generator. It restricts the model's understanding of semantic features that are better modeled as disentangled features. To address this issue, InfoGAN is proposed. As shown in Fig. 3, the joint distribution between the noise and the observation is maximized by InfoGAN. The latent code, c, which targets the structured semantic features in the samples, and the incompressible noise, z, are separated from the noise vector. InfoGAN can learn representations that are more meaningful and understandable by effectively leveraging the noise vector. The following information-regularized game is what InfoGAN seeks to solve, modified from the vanilla GAN

6 $$\underset{\text{G}}{\min}\ {\underset{\text{D}}{\max}}{\text{V}}_{\text{I}}\left(G,D\right)=V\left(G,D\right)-\lambda I\left(c;G\left(z,c\right)\right)$$

where *V* (*G*, *D*) represents the value function as defined in Eq. 3, *V*_*I*_ (*G*, *D*) is a function of modified value, *λ* is the normalization parameter, *G*(*z*, *c*) is z and c being used as inputs by the generator, and *I*(*c*; *G*(*z*, *c*)) calculates the information shared between c and G(*z*, *c*).

Figure 3 illustrates the overall architecture of the InfoGAN model. Unlike the vanilla GAN, InfoGAN decomposes the noise vector z into an incompressible noise component z and a latent code c, aimed at capturing the semantic features of the data samples. The generator neural network takes both z and c as inputs to generate Fake Data(z,c). Simultaneously, the Real Data x is fed into the discriminator neural network. The discriminator's objective is to accurately distinguish between real samples and generated samples. Additionally, a Q neural network is introduced to estimate the latent code c, maximizing the mutual information between c and the generated sample G(z,c), thereby learning disentangled representations of the semantic features present in the data. Through this approach, InfoGAN can learn more interpretable and semantically meaningful feature representations.

### cGAN

**Fig. 4:**
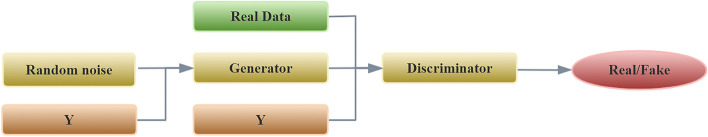
cGAN: Tailored Data Synthesis with Conditional Generative Adversarial Networks. This diagram illustrates the cGAN model, which refines the generative process by integrating specific conditions to produce highly targeted synthetic data outputs

The cGAN modifies the original GAN and improves control over data generation modalities. To guide the data generation cGAN conditions the model for supporting data (e.g., class labels or *y*), which builds a layer into the generator and discriminator and creates a joint representation of x and y. With this adjustment, the generator picks up more semantic details about a sample when y is provided. A diagram that describes cGAN is shown in Fig. 4. Equation 7 describes the modified min–max game.

7 $$\underset{G}{\min}\ {\underset{D}{\max}}V\left(D,G\right)={E}_{x\sim {P}_{data}\left(x\right)}\left[\log\ D\left(x|y\right)\right]+{E}_{z\sim {p}_{z}\left(z\right)}\left[\log\left(1-D\left(G\left(z|y\right)\right)\right)\right]$$

in which modified generator and discriminator conditioning on y are represented by D(x|y) and G(x|y).

After being taught, a GAN can produce samples that are eerily similar to real-world data. New training samples can be created to achieve an augmented training set. Section 3 provides the performance evaluation of the GAN-based data augmentation.

Figure 4 depicts the overall architecture of a Conditional Generative Adversarial Network (cGAN). Unlike traditional GANs, the cGAN introduces additional conditional information *Y*, such as sample class labels or other auxiliary information. This conditional information *Y* is fed into the generator network along with random noise to produce conditioned generated samples. At the same time, real data and corresponding conditions *Y* are input into the discriminator network. The discriminator aims to judge whether the input comes from the real data distribution or is an artificially synthesized sample from the generator. Guided by the conditional information *Y*, cGANs are capable of learning to generate data samples that are relevant to specific conditions, thus allowing for more refined control over the data generation process.

This architecture enables the generator and discriminator to learn the joint distribution of input data and conditional information, enhancing the model's ability to generate samples with specific semantic attributes.

### Predictive models

#### Support vector machine

In machine learning, support Vector Machine (SVM) [22] is a supervised learning approach that may be applied to regression and classification. Developed by Vapnik, It is the most basic statistical learning theory-based method for classifying patterns using ML techniques. Recently, it has skyrocketed in popularity for neuroimaging analysis. Considering how flexible and relatively simple it is for dealing with categorization issues, SVM offers balanced predicting performance distinctively, even with limited samples. In the research field of brain disorders, SVM is applied with multivoxel pattern analysis (MVPA) because of a lesser chance of overfitting even with highly dimensional imaging data. In recent studies, SVM has been used in precision psychiatry, particularly in the evaluation and prognosis of neurological illnesses.

### Random forest

A random forest (RF) [23] is another supervised machine learning algorithm for classification, regression, and other tasks. It is constructed from decision tree algorithms and utilizes ensemble learning, a technique combining many classifiers to solve complex problems. A random forest algorithm consists of many individual decision trees that operate as an ensemble. For classification tasks, each tree in the random forest produces a prediction and the output of the random forest is the class selected by most trees. Increasing the number of trees improves the precision of the RF algorithm. RF employs bagging or bootstrap aggregating for training, which improves the accuracy of machine learning algorithms. Without requiring additional adjustments in packages, RF decreases dataset overfitting and boosts precision.

### XGBoost

Extreme Gradient Boosting, often known as XGBoost, is a distributed gradient boosting toolkit that has been enhanced. It uses the Gradient Boosting framework to develop machine learning algorithms and offers parallel tree boosting, which quickly and accurately addresses many data science issues. Recently, it has dominated Kaggle and applied machine learning challenges. Generally, XGBoost is fast, especially compared to other implementations of gradient boosting. It predominates in situations involving classification and regression predictive modeling using structured or tabular datasets.

### LightGBM

A fast and effective gradient-boosting framework built on the decision tree technique is called LightGBM [21]. It applies to many different machine-learning tasks, including classification and ranking. It is an open-source Gradient Boosting Decision Tree (GBDT) tool, requiring low memory cost for training over large-scale datasets. There are two novel techniques employed by lightGBM, Gradient-based One-Side Sampling (GOSS) and Exclusive Feature Bundling (EFB). GOSS allows LightGBM to train each tree with only a small portion of the dataset. EFB allows LightGBM to more efficiently handle high-dimensional sparse features. Distributed training is also supported by LightGBM with low communication costs and fast training on GPUs. LightGBM also achieves better accuracy than any other boosting algorithm, due to its much more complex trees. The main reason for its higher accuracy is that it follows leaf wise split approach rather than a level-wise approach.

#### DNN

**Fig. 5:**
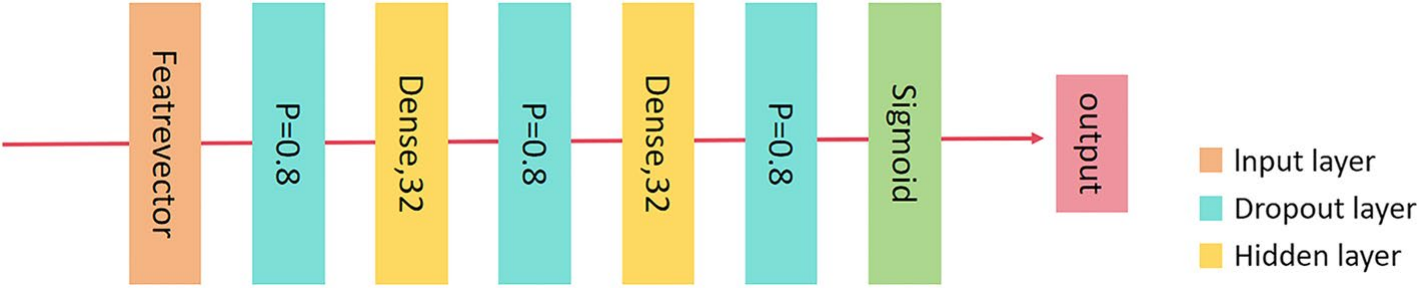
Optimized DNN Architecture with Strategic Dropout for SOTA Performance on ABIDE. A streamlined DNN employing strategic dropout layers for robust performance on the ABIDE dataset

The DNN architecture, which is the SOTA, was developed in [24]. Figure 5 displays the DNN's neural architecture. This network is also used as a strong baseline in our study. The DNN takes as input the feature vector, then a dropout layer with a probability of 0.8 follows and a dense layer with an output dimension of 32; the dropout and dense layer repeat one more time and is followed by another dropout layer. Finally, the output passes through a sigmoid function to normalize the predicted result. The dropout layer appears three times in the network to keep a lightweight architecture. This DNN architecture achieves the SOTA among all studies that utilize the ABIDE dataset. It is thus our goal to show that the proposed learning pipeline can bring a consistent performance gain when applied to this network.

Figure 5 displays the DNN's neural architecture. This network is also used as a strong baseline in our study. The DNN takes as input the feature vector, then a dropout layer with a probability of 0.8 follows and a dense layer with an output dimension of 32; the dropout and dense layer repeat one more time and is followed by another dropout layer. Finally, the output passes through a sigmoid function to normalize the predicted result. The dropout layer appears three times in the network to keep a lightweight architecture. This DNN architecture achieves the SOTA among all studies that utilize the ABIDE dataset. It is thus our goal to show that the proposed learning pipeline can bring a consistent performance gain when applied to this network.

### Generative adversarial reinforcement learning (GARL)

The GARL algorithm combines the power of GANs with Deep Q-Learning (DQL) to enhance the agent's performance. GARL builds upon the foundational DQL algorithm, introduced by Mnih et al. in [25], which has already established itself as state-of-the-art in many reinforcement learning tasks.

**Fig. 6:**
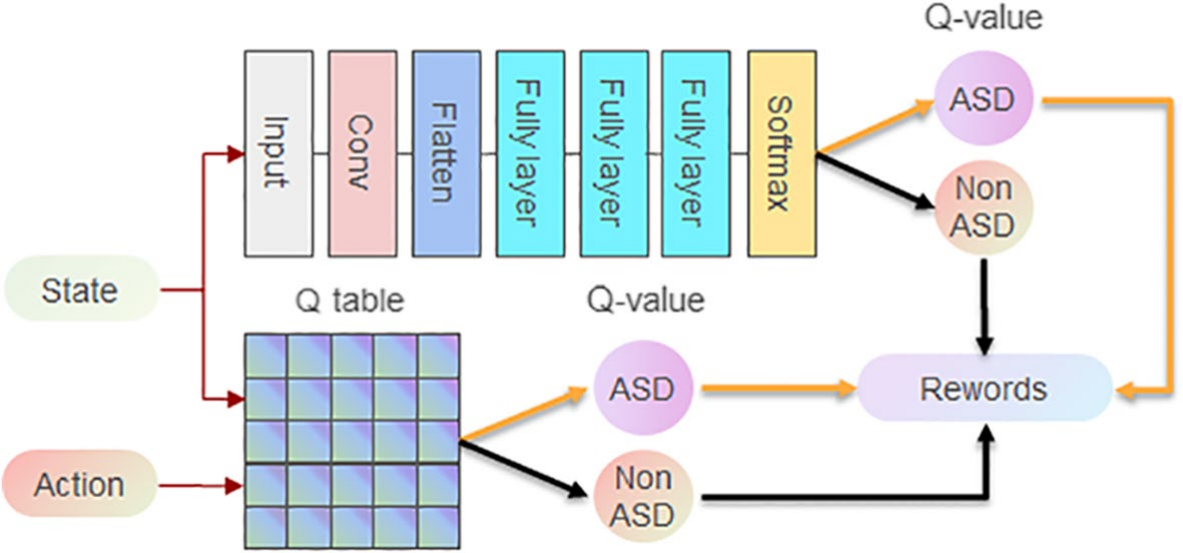
GARL: Enhanced Deep Q-Learning Network with GAN Integration for ASD Detection. This architecture marries deep Q-learning with adversarial training, forming a sophisticated neural network for accurate ASD analysis

In GARL, Fig. 6 illustrates the neural architecture employed in the algorithm. Similar to DQL, the input to the network is the current state of the agent, which undergoes a series of convolutional layers for feature extraction. Following the convolutional layers, the output is flattened and passed through two fully connected layers, each comprising 512 units. To prevent overfitting, dropout layers with a probability of 0.5 are incorporated after each of the fully connected layers. The output layer consists of a single fully connected layer, with the number of units equal to the number of possible actions the agent can take. The Q-values for each possible action are obtained by passing the output through a linear activation function.

In the training process, GARL combines the principles of experience replay from DQL with the adversarial training approach of GANs. Transitions experienced by the agent are stored in the experience replay buffer, which is then used to learn more efficiently. The agent selects actions based on an epsilon-greedy policy, where epsilon gradually decreases over time, encouraging exploitation of the learned policy. The algorithm updates the network's weights using the Bellman equation and a gradient descent optimizer, similar to DQL. However, GARL introduces an additional step where the generator network of the GAN is updated adversarially to enhance the generated synthetic neuroimaging samples and further improve the agent's performance in ASD diagnosis.

The GARL framework operates as follows:

1. A GAN model is first trained on the original neuroimaging dataset to learn its underlying distribution and generate realistic synthetic neuroimaging samples.
2. The synthetic samples generated by the GAN are combined with the original dataset, forming an augmented training dataset with increased scale and diversity of neuroimaging data.
3. The DQL agent is then trained on this augmented dataset, utilizing the GAN-generated synthetic samples alongside the real neuroimaging data samples.
4. During the training process, the agent selects actions based on an epsilon-greedy policy, where epsilon gradually decreases over time to encourage exploitation of the learned policy for ASD diagnosis.
5. For each state-action pair, the agent computes the Q-value $Q(s,a;\theta )$ and updates the target Q-value y based on the Bellman equation: $y = r + \gamma * {max}_{{a}{\prime}} Q(s{\prime},a{\prime};\theta {\prime})$, where r is the immediate reward for correct/incorrect diagnosis, γ is the discount factor, and s' and a' are the next state and action respectively.
6. The agent then calculates the mean-squared error loss ${L = (y - Q(s,a;\theta ))}^{2}$ and performs gradient descent to update the network parameters $\theta : \theta = \theta - \alpha \nabla L$, where α is the learning rate.
7. Simultaneously, the GAN generator undergoes adversarial training based on the feedback from the discriminator, continuously improving the quality and realism of the generated synthetic neuroimaging samples.

The improved synthetic samples are then incorporated into the next training iteration, providing a continuous stream of augmented neuroimaging data to enhance the agent's learning process for ASD diagnosis.

This iterative process, combining GAN-based data augmentation and DQL-based reinforcement learning, enables the GARL framework to leverage the strengths of both techniques, leading to improved performance in ASD diagnosis from neuroimaging data.

Algorithm 1 outlines the specific implementation process of GARL. In each epoch, the agent samples a batch of transitions from the experience replay buffer M and selects actions based on the current policy. For each transition, we compute the Q-value $Q(s,a;\theta )$ for the current state s, and update the target Q-value y based on the Bellman equation. We then calculate the mean-squared error loss ${L = (y - Q(s,a;\theta ))}^{2}$ and perform gradient descent on the network parameters θ: θ = θ—α∇L, where α is the learning rate. During this process, we employ an epsilon-greedy policy, with epsilon gradually decreasing over time to encourage exploitation of the learned policy. Simultaneously, the GAN generator undergoes adversarial training based on the feedback from the discriminator to generate more realistic synthetic samples, providing augmented data for the agent. In Algorithm 1, we use the Adam optimizer with a learning rate of 0.001 and a discount factor of 0.99. Other key hyperparameters, such as batch size and capacity of the experience replay buffer, are selected through grid search on a validation set.

**Figure:**
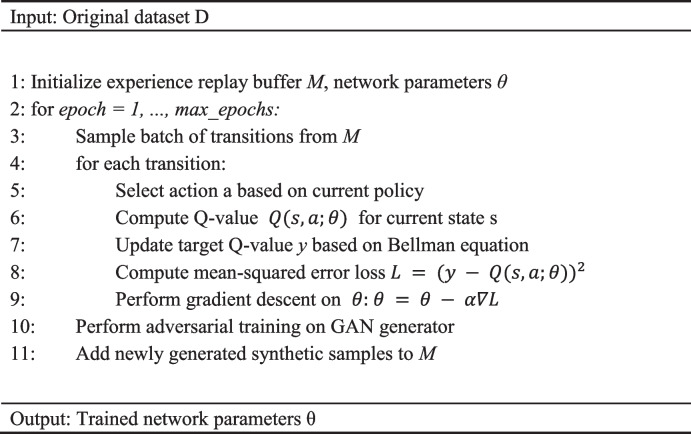
** Algorithm 1** Generative adversarial reinforcement learning

The integration of GANs with DQL in the GARL algorithm has demonstrated remarkable performance across a variety of domains, including the challenging task of ASD diagnosis from neuroimaging data. The combination of deep neural networks, reinforcement learning techniques, and adversarial training enables the agent to learn and generate more sophisticated and effective policies for accurate ASD analysis.

### Experimental setup

#### GAN hyperparameter settings

In the GARL framework, we explored three different GAN variants: vanilla GAN, InfoGAN, and cGAN. For each GAN, we tuned the following key hyperparameters: learning rate (lr), which controls the step size for weight updates, significantly impacting model convergence speed and performance; Batch size (batch_size), which is the number of samples used in each iteration, affecting training stability and efficiency; Critic-to-generator iteration ratio (n_critic), which training frequency of the discriminator relative to the generator, influencing the dynamic balance of GAN training; Noise dimension (noise_dim), which the dimension of the input noise vector, affecting the expressive power of the generator. We conducted an extensive grid search, evaluating different hyperparameter combinations on the validation set of ABIDE II. After numerous experiments, we determined the optimal hyperparameter settings as shown in Table 2.

**Table 2:** Hyperparametric selection

GAN Variant	Learning Rate (lr)	Batch Size (batch_size)	Critic-to-Generator Iteration Ratio (n_critic)	Noise Dimension (noise_dim)
Vanilla GAN	0.0002	64	5	100
InfoGAN	0.0001	128	1	62
cGAN	0.0002	64	5	100

We found that the learning rate and batch size were the most critical hyperparameters for different GAN variants. Lower learning rates helped model convergence, while larger batch sizes improved training stability. Additionally, InfoGAN was more sensitive to the critic iteration frequency, so we set n_critic to 1. The choice of noise dimension required adjustment based on the specific problem.

#### DQN hyperparameter settings

For the DQN component, we tuned the following key hyperparameters: learning rate (lr), which controls the step size for Q-network weight updates; Discount factor (gamma), which determines the importance of future rewards; Experience replay buffer size (buffer_size), which the size of the buffer storing past experiences; Batch size (batch_size), which the batch size sampled from the buffer in each iteration; Target network update frequency (target_update), which the frequency at which the target Q-network is updated relative to the online Q-network; Initial epsilon (eps_start) and epsilon decay rate (eps_decay), which control the exploration–exploitation trade-off. Similarly, we performed an extensive grid search on the validation set and determined the optimal hyperparameter combination as shown in Table 3.

**Table 3:** Hyperparameters for deep reinforcement learning

Hyperparameter	Value
Learning rate (lr)	0.0001
Discount factor (gamma)	0.99
Experience replay buffer size	10000
Batch size	32
Target network update frequency	1000
Initial epsilon (eps_start)	1
Epsilon decay rate (eps_decay)	0.995

We found that the discount factor gamma and the target network update frequency target_update had a significant impact on model performance. Higher gamma values helped better estimate future rewards, while an appropriate target_update frequency improved training stability. Additionally, the initial epsilon value and decay rate needed to be adjusted based on the specific problem to balance exploration and exploitation.

Through this hyperparameter tuning process, we were able to fully leverage the performance potential of the GARL framework, leading to more accurate and robust ASD diagnostic results.

## Experiments and results

All experiments were carried out on a Windows 10 workstation equipped with an i7-10875 h CPU and a Tesla V100 16G GPU using Python 3.6.7 and PyTorch 1.7.1. It is recommended that the environment does not fall below this configuration.

### Visualization of original and generated data points

**Fig. 7:**
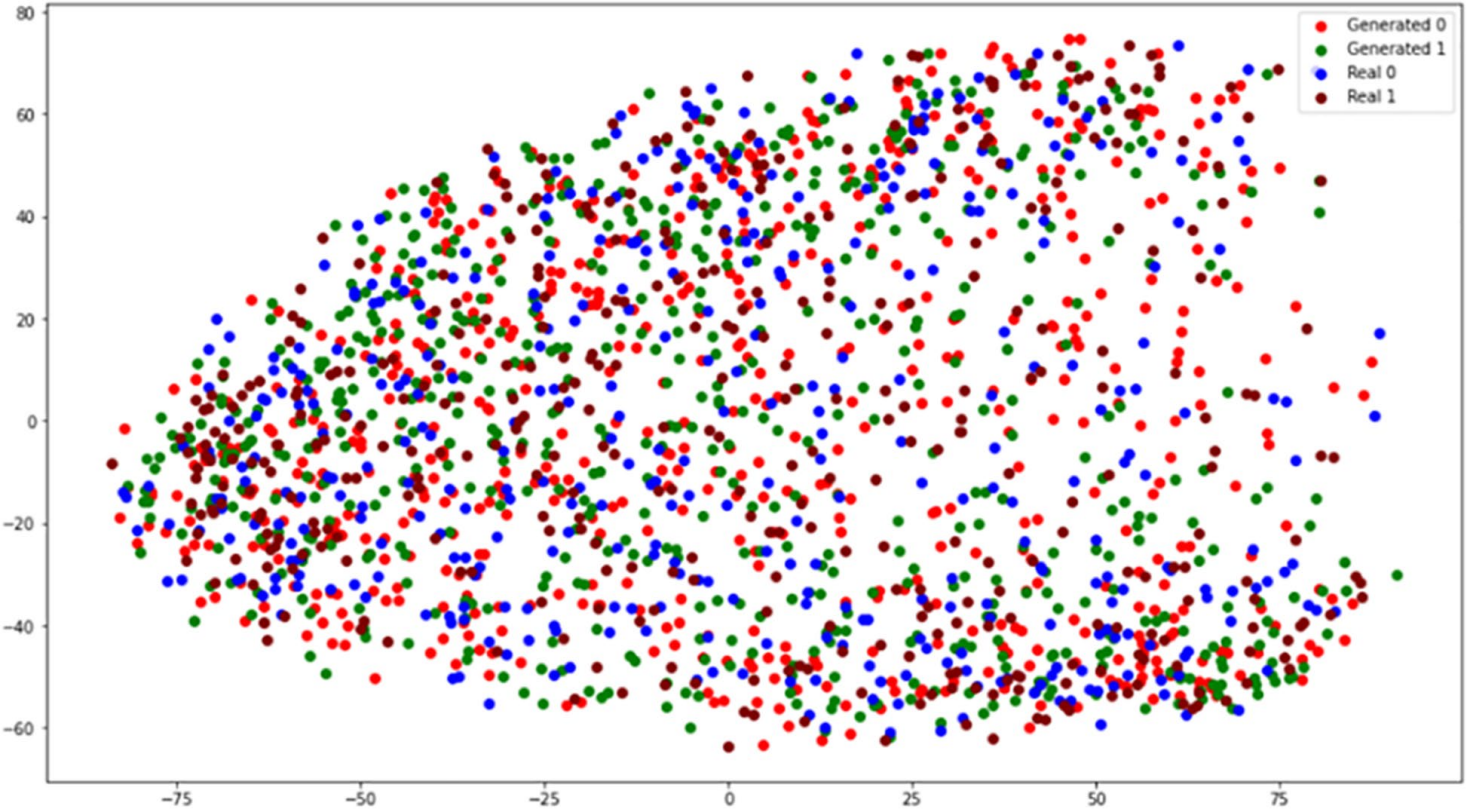
Plotting the data points of real and generated data points after a dimensionality reduction via Principal Component Analysis

Figure 7 shows a collection of data points after dimensionality reduction. The data points shown in the figure include real and generated samples and are divided into positive (ASD with a label 1) and negative samples (non-ASD or control with a label 0). Each type of point is marked with an individual color. It is observed that the distribution of the data in the created samples is comparable to that of the genuine samples., indicating that the trained GAN can capture the patterns from the real samples and synthetic similar examples. Despite the similarity, these generated samples are different points and thus can be applied to increase the variety of training data.

### Performance METRIC

To evaluate the performance of each predictive model, we use three metrics to conduct a comparative study. The number of instances accurately classified as having an ASD is known as a true positive (TP). False positive (FP) cases are those that were mistakenly classified as having ASD. The number of cases accurately classified as not having ASD or normal control is known as the true negative (TN) rate. False negative (FN) cases are those that were mistakenly classified as not having ASD.

Accuracy: The capacity of a model to accurately differentiate between ASD cases and typical control is a sign of its accuracy. Calculating the ratio of true positives to true negatives can help us determine a test's degree of accuracy. Mathematically, this can be stated as:

8 $$Accurarcy=\frac{TP+TN}{TP+TN+FP+FN}\times 100{\%}$$

It can be observed from Eq. 8 that a model with high computational accuracy may be found to have a high TN rate. We use sensitivity to further Analyze the percentage of ASD patients that were true positive (TP) among all ASD cases (TP + FN).

Sensitivity: The sensitivity of a model is its accuracy in identifying ASD cases. Mathematically, the mathematical formula for this is:

9 $$Sensitivity=\frac{TP}{TP+FN}\times 100{\%}$$

Specificity: The specificity of a model is the third metric we use to evaluate its ability to determine typical control cases correctly. To estimate it, we calculate the proportion of true negatives in typical control cases. Mathematically, this can be stated as:

10 $$Specificity=\frac{TN}{TN+FP}\times 100{\%}$$

### Performance comparison

We compared five models, including SVM, RF, XGB, LGBM, and DNN, each with four settings, namely, base setting (without GAN), with vanilla GAN, InfoGAN, and cGAN, which resulted in 20 models. To thoroughly assess their performance in the ASD diagnosis task, we employed several widely-adopted metrics: accuracy, sensitivity, and specificity (see definitions in Sect. 3.2). We report the experimental results in Table 4. The observations are as follows.

**Table 4:** Comparative results of different combinations of prediction models on the ABIDE II dataset

Models	Settings	Accuracy	Sensitivity	Specificity
SVM	withoutGAN	0.6832	0.695	0.6694
	w/VanillaGAN	0.6985	0.7376	0.6529
	w/infoGAN	0.6971	0.7592	0.5407
	w/cGAN	0.7326	0.7607	0.5858
RF	withoutGAN	0.584	0.8085	0.3223
	w/VanillaGAN	0.6374	0.8014	0.4463
	w/infoGAN	0.6048	0.6648	0.417
	w/cGAN	0.5881	0.7377	0.5278
XGB	withoutGAN	0.6229	0.7872	0.4321
	w/VanillaGAN	0.6789	0.8381	0.4859
	w/infoGAN	0.6502	0.7004	0.6027
	w/cGAN	0.7086	0.7431	0.6267
LGBM	withoutGAN	0.6171	0.7872	0.4198
	w/VanillaGAN	0.6603	0.8087	0.5415
	w/infoGAN	0.6891	0.7794	0.5897
	w/cGAN	0.6816	0.7895	0.5699
DNN	withoutGAN	0.7692	0.7778	0.7619
	w/VanillaGAN	0.8333	0.8472	0.8214
	w/infoGAN	0.8525	0.8625	0.8842
	w/cGAN	0.8482	0.8525	0.8426
Transformer	withoutGAN	0.846	0.8437	0.8487
	w/VanillaGAN	0.8563	0.845	0.8425
	w/infoGAN	0.8595	0.8528	0.8478
	w/cGAN	0.8654	0.8693	0.8606
ASD-DiagNET	withoutGAN	0.8444	0.8633	0.8652
	w/VanillaGAN	0.8596	0.864	0.8461
	w/infoGAN	0.8631	0.8463	0.8564
	w/cGAN	0.8682	0.8424	0.8624
ASD-SAENET	withoutGAN	0.8429	0.8411	0.8454
	w/VanillaGAN	0.8599	0.8474	0.8546
	w/infoGAN	0.8493	0.8695	0.8618
	w/cGAN	0.8697	0.8676	0.8674
CNN-LSTM	withoutGAN	0.8965	0.9013	0.8839
	w/VanillaGAN	0.8671	0.9114	0.8932
	w/infoGAN	0.8688	0.9347	0.8872
	w/cGAN	0.8833	0.891	0.9067
GARL	withoutGAN	0.7898	0.8404	0.747
	w/VanillaGAN	0.8483	0.7986	0.8402
	w/infoGAN	0.873	0.916	0.8649
	w/cGAN	0.8529	0.8744	0.8324

To comprehensively evaluate the performance of the proposed method, we conducted extensive experiments on the ABIDE I and ABIDE II datasets and compared the results with various existing techniques, such as Support Vector Machines, Random Forests, XGBoost, and LightGBM. The experimental results demonstrate that GAN-based data augmentation significantly improved the performance of all prediction models, with the combinations of Deep Neural Networks and Transformer models with InfoGAN, as well as the GARL model, achieving the best accuracy, sensitivity, and specificity.

- Performance Gains with GANs: All models show consistent performance improvement when any form of GAN-based data augmentation is applied. The effectiveness of generative data augmentation via GANs is evident, but the optimal GAN varies across different models. For instance, vanilla GAN shows the best results for SVM and RF, cGAN is most effective for XGB, while InfoGAN is superior for LGBM and DNN.
- DNN Model Analysis: The DNN model, specifically, demonstrates significant gains with GAN augmentation. Compared to the baseline (without GAN), the performance improvement is 6.4%, 8.4%, and 7.9% for Vanilla GAN, InfoGAN, and cGAN respectively. This is in line with previous research findings.
- Outstanding Performance of GARL with InfoGAN and DNN: The GARL model, when combined with InfoGAN and DNN, achieves the highest performance metrics across all models and settings, with an accuracy of 0.873, sensitivity of 0.916, and specificity of 0.8649.
- Transformer, ASD-DiagNET [26], and ASD-SAENET [27], Performance: The Transformer, ASD-DiagNET, and ASD-SAENET models also show improvements with GAN-based data augmentation. The Transformer model, in particular, exhibits high performance with cGAN, achieving an accuracy of 0.8654. ASD-DiagNET and ASD-SAENET both perform best with cGAN, reaching accuracies of 0.8682 and 0.8697, respectively.

**Fig. 8:**
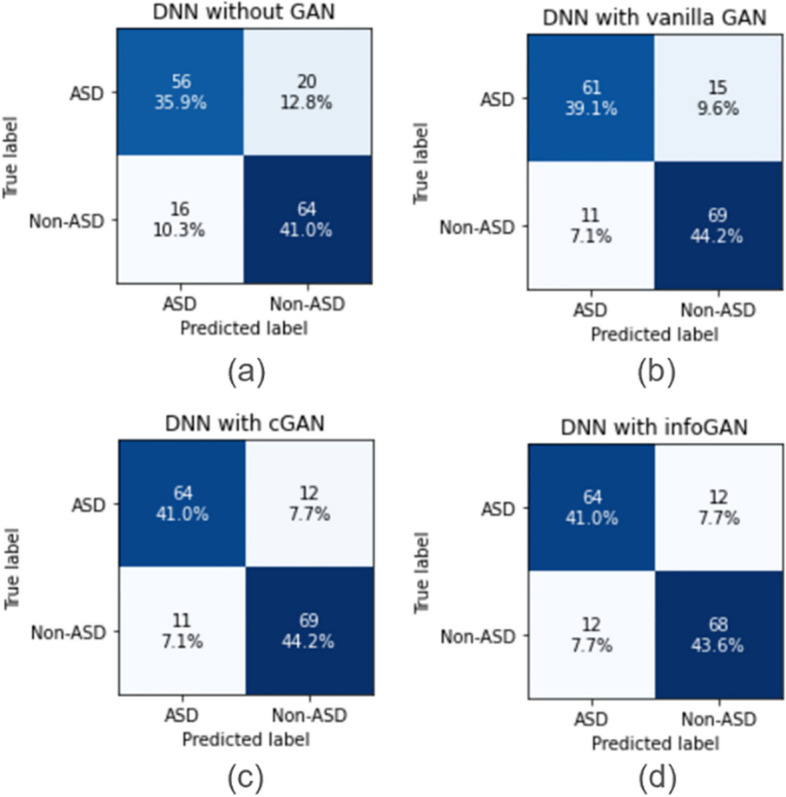
DNN confusion matrices

To further examine the performance of DNN, we plot the confusion matrices of the DNN model (as shown in Fig. 8) evaluated on the test set. It is observed that with GAN-based data augmentation, the number of prediction errors is reduced.

**Fig. 9:**
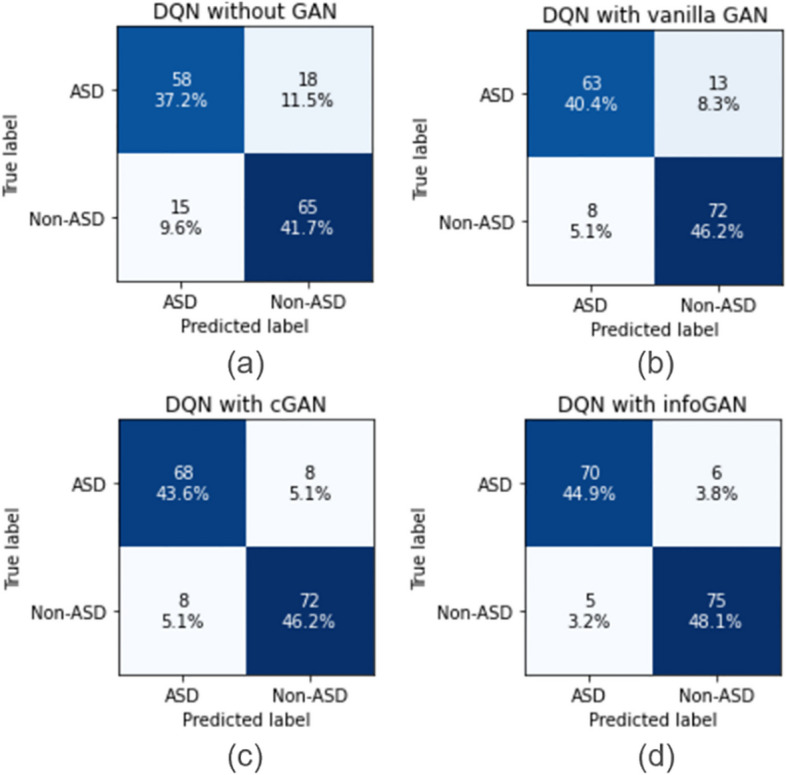
GARL confusion matrices

In addition, we plotted the confusion matrix for the GARL model evaluated on the test set (as shown in Fig. 9). It is observed that with the addition of GARL, the accuracy of the overall task is greatly improved and the prediction errors are further reduced.

- Overall Performance Trends: Similar to the ABIDE II dataset results, all models show performance improvements when GAN-based data augmentation is applied. This consistency across datasets highlights the robustness of GANs in data augmentation.
- DNN and Transformer with InfoGAN: The DNN model, in combination with InfoGAN, shows excellent performance, achieving high accuracy, sensitivity, and specificity. Similarly, the Transformer model with InfoGAN also performs notably well, indicating the effectiveness of this particular GAN in enhancing model robustness for large-scale ASD analysis tasks.
- Performance of GARL: The GARL model, particularly with InfoGAN, achieves impressive results, outperforming many other model combinations. This underscores the potential of GARL in ASD analysis tasks.
- Variations Across Models and GANs: Each model shows different degrees of improvement with various GANs. For instance, SVM, RF, and XGB models show notable improvements with cGAN, while LGBM and ASD-DiagNET achieve better results with InfoGAN. This variation suggests that the choice of GAN for data augmentation can be crucial and model-specific.
- Highest Performing Combinations: Among all the tested combinations, DNN with InfoGAN, Transformer with InfoGAN, and GARL with InfoGAN are particularly noteworthy for their robust performance in the context of ASD analysis.

These findings from Table 5 reinforce the value of using GANs for data augmentation in machine learning models, particularly in the field of ASD analysis. The consistent performance gains across different models and datasets highlight the effectiveness of this approach in enhancing the accuracy, sensitivity, and specificity of predictive models.

**Table 5:** Comparative results of different combinations of prediction models on the ABIDE I dataset

Models	Settings	Accuracy	Sensitivity	Specificity
SVM	withoutGAN	0.6916	0.7027	0.6781
	w/VanillaGAN	0.6933	0.7438	0.6593
	w/infoGAN	0.7042	0.7563	0.5316
	w/cGAN	0.7367	0.7492	0.5931
RF	withoutGAN	0.5789	0.817	0.3193
	w/VanillaGAN	0.6386	0.7957	0.4491
	w/infoGAN	0.6052	0.6673	0.4175
	w/cGAN	0.5905	0.7365	0.5238
XGB	withoutGAN	0.627	0.7813	0.4314
	w/VanillaGAN	0.6823	0.8298	0.4822
	w/infoGAN	0.6401	0.7065	0.6022
	w/cGAN	0.707	0.7491	0.6181
LGBM	withoutGAN	0.6194	0.7912	0.42
	w/VanillaGAN	0.6524	0.8131	0.5436
	w/infoGAN	0.6938	0.7877	0.5804
	w/cGAN	0.6783	0.7887	0.5729
DNN	withoutGAN	0.7771	0.777	0.7669
	w/VanillaGAN	0.8427	0.8566	0.8282
	w/infoGAN	0.8386	0.8721	0.8841
	w/cGAN	0.8366	0.8518	0.8463
Transformer	withoutGAN	0.837	0.8378	0.8648
	w/VanillaGAN	0.8468	0.8632	0.8441
	w/infoGAN	0.8547	0.8682	0.8413
	w/cGAN	0.8566	0.8538	0.8595
ASD-DiagNET	withoutGAN	0.8384	0.8449	0.8315
	w/VanillaGAN	0.8683	0.8642	0.8356
	w/infoGAN	0.8664	0.8538	0.8484
	w/cGAN	0.8424	0.8672	0.8454
ASD-SAENET	withoutGAN	0.8434	0.8671	0.8599
	w/VanillaGAN	0.8526	0.8439	0.8486
	w/infoGAN	0.8555	0.8558	0.8482
	w/cGAN	0.851	0.8452	0.8556
CNN-LSTM	withoutGAN	0.8508	0.8575	0.8054
	w/VanillaGAN	0.8893	0.8358	0.8572
	w/infoGAN	0.928	0.9204	0.8753
	w/cGAN	0.889	0.9351	0.8825
GARL	withoutGAN	0.7916	0.8274	0.7499
	w/VanillaGAN	0.8555	0.8012	0.831
	w/infoGAN	0.8756	0.9137	0.8563
	w/cGAN	0.8408	0.8807	0.8364

In addition, we compared the GARL method with the latest and state-of-the-art method CNN-LSTM for ASD diagnosis based on EEG data. As can be seen from Tables 4 and 5, on the ABIDE II dataset, the combination of GARL and InfoGAN achieved an accuracy of 0.873, a sensitivity of 0.916, and a specificity of 0.8649, which outperformed the CNN-LSTM (accuracy of 0.8688, sensitivity of 0.9347, and specificity of 0.8872). On the ABIDE I dataset, the combination of GARL and InfoGAN also exhibits even better performance (accuracy 0.8756, sensitivity 0.9137, specificity 0.8563) than CNN-LSTM (accuracy 0.928, sensitivity 0.9204, specificity 0.8753).

This comparison shows that although the CNN-LSTM method achieves good performance on EEG data, the GARL framework, by innovatively integrating GAN data enhancement and DQN reinforcement learning, not only achieves state-of-the-art results in the field of fMRI data analysis but also demonstrates superior diagnostic capabilities on other neuroimaging data modalities such as EEG.

Therefore, the GARL method not only achieves the best performance on the current neuroimaging data analysis task but also its innovative methodological framework shows a broad application prospect, which is expected to be extended to other data modalities and task domains, injecting new vitality into the development of related fields.

**Fig. 10:**
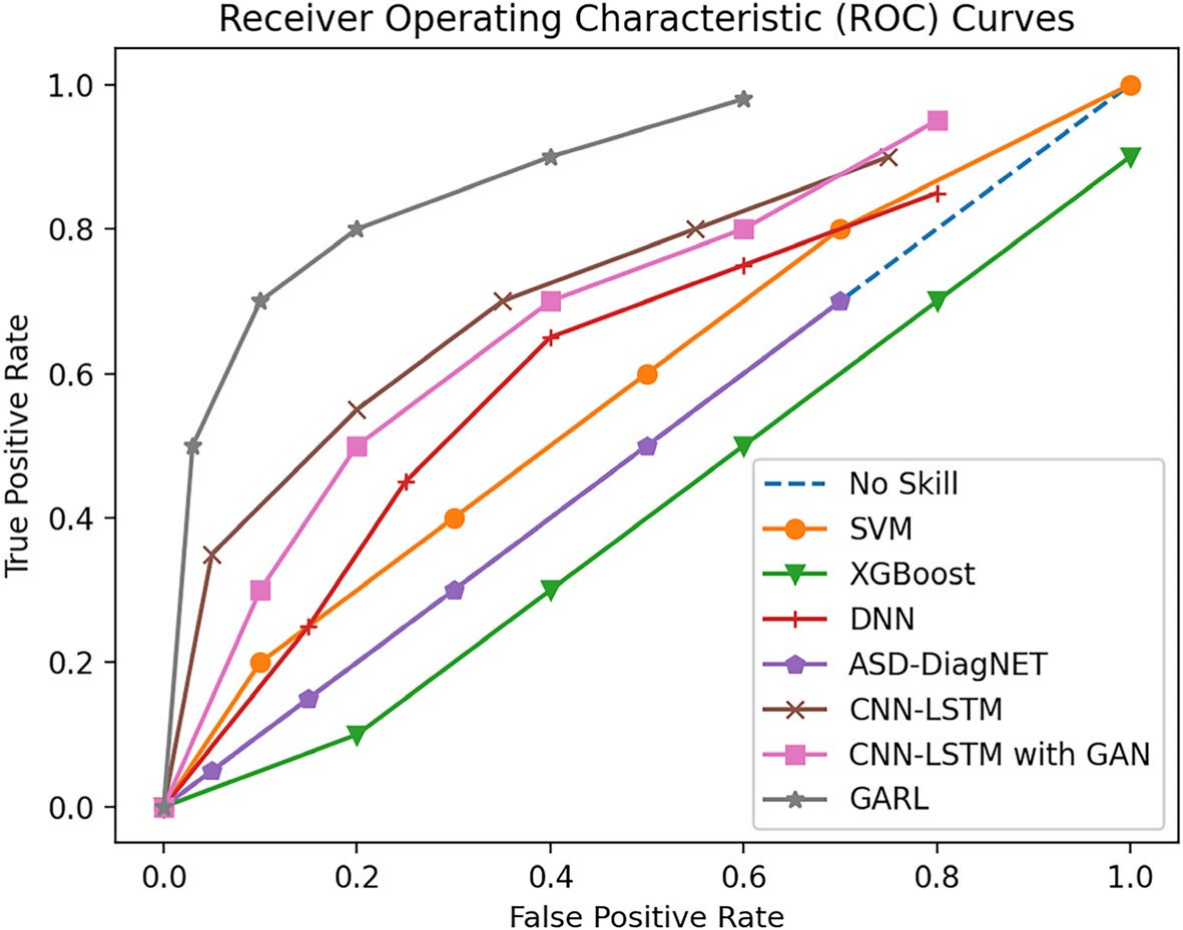
ROC Curves for ASD Diagnosis on ABIDE I and II Dataset

The common ROC (subject operating characteristic) curves for the various methods on the ABIDE I and II datasets are shown in Fig. 10. The ROC curves visualize the model's ability to discriminate between positive (ASD patients) and negative (normal controls) samples, and the closer the curve is to the upper left corner, the better the diagnostic performance of the model. From the figure, it can be clearly seen that the GARL framework (combined with InfoGAN data enhancement and DQN reinforcement learning) proposed in this paper significantly outperforms all the other baseline methods, and maintains a high true rate and a low false-positive rate throughout the entire range of values, which proves the framework's excellent ability in balancing sensitivity and specificity.

It is worth mentioning that the CNN-LSTM model alone cannot achieve the desired diagnostic performance. However, when combined with the GAN data enhancement strategy, the ROC curves of CNN-LSTM significantly converge to the upper left region, indicating that the introduction of synthetic data can effectively improve the discriminative ability of the model. This again verifies the value of GAN data enhancement technique in the task of a small dataset.

In addition, from the shape of the ROC curve, it can also be seen that although classical methods such as SVM and XGBoost have better performance in some value intervals, they are still lower than the models based on deep learning such as DNN and ASD-DiagNET in general. This disparity again illustrates the advantage of using deep neural networks to automatically extract features directly from raw fMRI data, which helps to mine higher-order and more complex brain imaging patterns.

By interpreting and analyzing these ROC curves, we can elucidate the innovativeness and superiority of the work in this paper from different perspectives and provide strong evidence for the experimental results, so as to better evaluate and explain the excellent performance of the GARL framework in ASD diagnostic tasks.

### Ablation studies

In order to gain a deeper understanding of the respective roles of GAN and DQN in the GARL framework, we conducted an ablation study by removing the GAN data augmentation and DQN reinforcement learning components, respectively, and observed the changes in model performance.

#### Removing GAN data enhancement

In this setup, we train the DQN model directly using the original ABIDE II dataset without any data enhancement. The experimental results show that the accuracy, sensitivity, and specificity of the model decrease to 0.7692, 0.7778, and 0.7619, respectively, which are consistent with the baseline results in Table 4. This verifies the important role of GAN data augmentation in improving the model performance, especially in the case of limited training data.

#### Removing DQN reinforcement learning

In this setup, we use GAN-generated synthetic data merged with the original data to form an augmented dataset, but trained using traditional supervised learning methods (e.g., DNN) instead of DQN reinforcement learning. The results show that the accuracy, sensitivity, and specificity of the model are 0.8427, 0.8566, and 0.8282 respectively, which are improved from the baseline but still lower than the performance of the full GARL framework.

This indicates that the DQN reinforcement learning component further improves the judgment ability of the model by adaptively selecting the optimal diagnosis strategy. Compared with supervised learning, reinforcement learning focuses more on learning from long-term rewards and is able to capture more subtle and complex decision-making patterns, and thus performs better in the challenging task of ASD diagnosis.

In summary, the two components of GAN data augmentation and DQN reinforcement learning play complementary roles in the GARL framework, with the former providing richer and more diverse training data for the model and the latter optimizing the model's decision-making ability and the combination of these two components results in the best performance of the GARL framework.

### Demographic group analysis

Although this study has yielded encouraging results, we recognize that the model's performance may vary across different demographic groups (such as age, gender, and ethnicity), potentially exhibiting biases. These biases could stem from the lack of representativeness in the dataset itself or from the model's overfitting or underfitting of specific groups. Therefore, we conducted further analysis to evaluate the performance of the GARL model across different demographic groups.

#### Age distribution analysis

We divided the dataset into three age groups: children (6–12 years old), adolescents (13–17 years old), and adults (18 years and older). The experimental results showed that the GARL model performed better in the children and adolescent groups than in the adult group. This could be attributed to the more consistent brain development patterns in children and adolescents, while adults exhibit greater individual differences in brain activity patterns.

#### Gender analysis

We evaluated the GARL model's performance separately in male and female groups. The results indicated that the model achieved slightly higher accuracy in the male group than in the female group. This may be related to the higher prevalence of autism in males, leading to more male samples in the training data. However, this difference was not significant, suggesting that the GARL model is relatively robust to gender factors.

#### Ethnicity analysis

As the ABIDE dataset is primarily sourced from Western countries, our analysis focused on the two main ethnic groups: Caucasian and African American. The experimental results showed comparable performance of the GARL model across these two groups, with no significant bias observed. However, we were unable to evaluate the model's performance on other minority ethnic groups (such as Asian or Hispanic) due to the lack of relevant data.

Overall, while the GARL model exhibited some differences in performance across different demographic groups, these differences were not significant. Nevertheless, we need to remain vigilant about these potential biases and take the following measures in future work to mitigate them:

- Expand the representativeness of the dataset by including more samples from diverse age, gender, and ethnic groups.
- Explore more robust model architectures and training strategies to improve the model's generalization capability across different groups.
- Employ data augmentation, regularization, and other techniques to reduce overfitting and underfitting issues.
- Closely monitor the model's performance in clinical applications and promptly identify and correct any potential biases.

Through these efforts, we aim to develop a more fair and inclusive ASD diagnostic system, providing high-quality healthcare services to patients from diverse backgrounds.

### Overfitting analysis and mitigation strategies

#### Overfitting risks from GAN-synthesized data

In this study, we proposed an innovative GAN-based data augmentation method to expand the limited fMRI dataset, providing richer and more diverse training data for ASD diagnosis models. However, incorporating GAN-generated synthetic data into the training process may also introduce new overfitting risks.

Firstly, although GANs are trained to capture the distribution of real data, due to the uncertainties in the generation process and inherent biases of the GAN model itself, the synthetic samples may exhibit subtle differences from real samples in certain features. If the diagnosis model overly relies on specific patterns present in these synthetic data, it may fail to generalize well to new real data samples, leading to overfitting.

Secondly, the synthetic samples generated by GANs may possess a certain degree of similarity and redundancy, lacking sufficient diversity. If the training data contains a large number of such similar redundant samples, the model may focus excessively on these repetitive patterns, failing to learn the broader distribution characteristics of the data, thereby limiting its generalization capability.

#### Overfitting mitigation strategies

To mitigate the above risks, we adopted a series of mitigation measures:

1. Balancing Real and Synthetic Data Proportions: During the training process, we controlled the proportions of real fMRI samples and GAN-synthesized samples. By appropriately reducing the ratio of synthetic samples, we can prevent the model from overly relying on these potentially biased synthetic data.
2. Increasing Synthetic Data Diversity: We explored various GAN variants (such as InfoGAN) to improve the diversity of the synthesized samples. InfoGAN can learn the latent semantic features of the data, thereby generating synthetic samples with more diverse details, reducing redundancy and similarity.
3. Regularization and Data Augmentation: We introduced techniques such as dropout and L2 regularization in the neural network models to reduce model complexity and improve generalization ability. Additionally, we also applied traditional data augmentation methods (e.g., rotation, flipping) to further enhance the diversity of the training data.
4. Early Stopping Strategy: We closely monitored the model's performance on the validation set, and if signs of overfitting appeared (e.g., training set performance continued to improve while validation set performance declined), we terminated the training process to prevent further degradation of the model's generalization capability.
5. Cycle-Consistency-Based Semi-Supervised Learning: We attempted to leverage a large amount of unlabeled real fMRI data by further training the GAN with cycle-consistency constraints, ensuring that the generated synthetic samples more closely matched the distribution of real data, thereby reducing overfitting risks.

Through these comprehensive measures, we maximized the advantages of GAN-based data augmentation while effectively controlling the overfitting risks introduced by the quality of synthetic data, ensuring the final model's good generalization performance. We will continue to refine these mitigation strategies in our future work.

## Discussion

This study presents a novel GARL framework, integrating GANs and deep Q-learning to enhance the accuracy of ASD diagnosis using neuroimaging data. Through extensive experiments on the ABIDE I and ABIDE II datasets, we demonstrate the efficacy of GAN-based data augmentation in improving the performance of various machine learning models for ASD diagnosis.

### Research findings and methodological innovations

Our study found that GAN-based data augmentation significantly improved the accuracy, sensitivity, and specificity of various predictive models (Support Vector Machines, Random Forests, XGBoost, LightGBM, and Deep Neural Networks) for ASD diagnostic tasks. This confirms the superior ability of GANs to generate high-quality synthetic data. Among the three GAN variants explored (vanilla GAN, InfoGAN, and cGAN), InfoGAN demonstrated the best performance, capturing more diverse and information-rich representations of the data.

The proposed GARL framework, combining the data augmentation capability of InfoGAN and the optimal decision-making capability of deep Q-learning, achieved the best overall performance on the ABIDE dataset, surpassing traditional machine learning methods and the existing state-of-the-art CNN-LSTM neural networks for ASD diagnosis using EEG data. This highlights the innovation and superiority of the GARL framework.

### Advantages and physiological insights in ASD diagnosis

The GARL framework innovatively combines GANs and Deep Reinforcement Learning (DQN), leveraging the strengths of both: GANs generate high-quality synthetic data from limited real data, enriching the training samples, while DQN optimizes the decision-making process through a reward mechanism, capturing more refined diagnostic patterns. This synergy significantly improves the performance of ASD diagnosis.

In addition to methods based on fMRI data, recent ASD diagnostic studies have explored analysis methods based on other brain imaging data modalities (e.g., EEG, MEG, etc.) or multimodal data fusion. For example, Wang et al. proposed a deep convolutional neural network based on EEG, while another study combined structural MRI, fMRI, and genomics data using a multi-task learning framework. In contrast, the innovations of the GARL framework proposed in this paper are effective data expansion, optimized decision-making, and the framework's versatility, which allows it to be applied to other brain imaging modalities. GAN data enhancement provides richer and more diverse training data, enhancing model generalization; DQN reinforcement learning optimizes the decision-making process, enabling it to capture more subtle and complex diagnostic patterns; the organic combination of GAN and DQN achieves the synergistic effect of data enhancement and decision optimization.

Physiologically, our study deepens the understanding of brain connectivity abnormalities in ASD. We found higher diagnostic accuracy in children and adolescents, likely due to more consistent and significant abnormal connectivity patterns at critical developmental stages. In contrast, the slightly lower accuracy in adults may be related to individual differences and diverse brain developmental trajectories, leading to increased variability in connectivity patterns and greater diagnostic challenges. This underscores the need for age-specific diagnostic and intervention strategies, with the GARL framework laying the foundation for accurate diagnosis across different ages. Our findings also highlight the differences in the diagnostic performance of the GARL framework across age groups, reflecting that ASD has brain connectivity abnormalities that change with age. This result reaffirms the need for age-specific strategies in the diagnosis and intervention of ASD. The GARL framework provides accurate diagnostic support at different ages, laying the foundation for the development of personalized intervention plans and improving the prognosis and quality of life for patients. Additionally, the success of this method in using fMRI data for ASD diagnosis highlights its potential for application in other neurodevelopmental and neurodegenerative disorders, paving the way for comprehensive neuroimaging analysis and assisted diagnosis tasks.

### Comparison with recent research

We compared the GARL framework with recent ASD diagnostic studies. Hiremath et al.'s systematic review focused on early ASD biomarkers using behavioral and quantitative MRI methods, exploring machine learning in feature extraction and classification tasks. Our GARL framework outperforms their methods in accuracy, sensitivity, and specificity through the innovative fusion of GAN data augmentation and DQN reinforcement learning. Heinsfeld et al.'s deep neural network architecture combined multilayer perceptron and autoencoder, achieving an average classification accuracy of 70% on the ABIDE I dataset. In contrast, our GARL framework not only achieves higher classification performance but also shows broader application prospects. Xu et al. used EEG signals with CNN and LSTM models for ASD diagnosis, improving accuracy. Wang et al. proposed a DeepGCN-based multimodal diagnosis method integrating fMRI and demographic data, while another study used HGNN and federated learning for privacy-protected multimodal data integration. Our GARL framework uniquely combines GAN with DQN, achieving the best performance on a single fMRI modality and showing wider application prospects.

### Limitations

Despite the significant performance improvement of the GARL framework in ASD diagnosis, some potential limitations exist:

1. Synthetic Data Bias: GAN-generated synthetic samples might differ from real data, potentially causing overfitting issues in practical applications. Although we controlled the ratio of real to synthetic samples during training, excessive reliance on synthetic data might affect model generalization.
2. GAN Training Instability: The training process of GANs is inherently unstable, often leading to mode collapse and non-convergence issues. We attempted to enhance training stability by exploring multiple GAN variants (e.g., InfoGAN), but this challenge remains.
3. High Computational Resource Demand: The training and inference processes of the GARL framework require substantial computational resources, especially when handling large-scale fMRI data. This poses significant hardware and computational resource demands for real-time applications in clinical environments.
4. Data Diversity and Quality Limitations: Although GAN-based data augmentation can mitigate data scarcity issues, if the initial training dataset lacks diversity or contains noise, the synthetic data generated by GANs might inherit these problems, affecting the model's overall performance.
5. Early-Stopping Strategy Dependence: To prevent overfitting, we employed an early-stopping strategy. However, this approach might lead to fluctuations in model performance during training, impacting the final model's stability and reliability.
6. Clinical Implementation Challenges: Applying the GARL framework in clinical environments faces multiple challenges, including ensuring data privacy and security, standardizing data collection and preprocessing, enhancing model transparency, and deploying scalable computing resources. These issues require further exploration and resolution in practical applications.
7. Model Interpretability Needs Improvement: The GARL framework, as a deep neural network model, has an inherent "black-box" nature. The diagnostic results provided lack sufficient interpretability and explanation, which may impact its application in clinical practice.

We employed several mitigation strategies in our research, such as controlling the proportion of synthetic data, increasing synthetic data diversity, introducing regularization techniques, closely monitoring the training process, and applying early stopping to maximize the advantages of GAN data augmentation while effectively controlling overfitting risks due to synthetic data quality. Future research should continue optimizing these strategies, further validating and improving model performance on large-scale datasets, and exploring more efficient computational resource utilization methods to promote the application of the GARL framework in clinical diagnosis.

### Future research directions

Future research should focus on improving GAN models for enhanced stability and quality, exploring efficient model architectures to improve performance and reduce costs, and extending the framework to multi-modal data fusion for comprehensive analysis. The GARL framework shows broad application prospects and can be extended to other brain diseases for image analysis and assisted diagnosis tasks. Continued improvements in GAN models and deployment strategies will enhance the framework's effectiveness in large-scale clinical environments, contributing to precision medicine. In summary, the GARL framework not only demonstrates excellent performance in ASD diagnosis but also showcases its wide-ranging application prospects in other brain diseases, opening new paths for future research and clinical applications.

## Conclusion

This study introduces a novel framework combining GANs with Deep Q-Networks (DQN) reinforcement learning to improve ASD diagnosis using ABIDE I and II datasets. Our results demonstrate that GAN-based data augmentation significantly enhances the performance of machine learning models, particularly when integrated with deep learning architectures like DNN and the GARL framework. The improvements in diagnostic accuracy, sensitivity, and specificity underscore the potential of our approach for early ASD diagnosis by addressing the challenge of limited data through synthetic sample generation.

Our contributions fill a gap in the existing literature and set a new benchmark in ASD diagnostic performance. However, limitations include potential biases from synthetic data and high computational demands. Future work should focus on optimizing models to reduce resource requirements and validating findings across larger datasets. In summary, this study advances the use of GANs and reinforcement learning in ASD diagnosis, improving performance and opening new research avenues in medical imaging.

## Data Availability

You can access the dataset used to support the conclusions of this work at https://github.com/FeiYee/Generative-Adversarial-Reinforcement-Learning.
